# Powassan Virus Infections: A Systematic Review of Published Cases

**DOI:** 10.3390/tropicalmed8120508

**Published:** 2023-11-26

**Authors:** Loukas Kakoullis, Victor Renault Vaz, Divmehar Kaur, Sonia Kakoulli, George Panos, Lin H. Chen, Irmgard Behlau

**Affiliations:** 1 Department of Medicine, Mount Auburn Hospital, Cambridge, MA 02138, USA; 2 Harvard Medical School, Boston, MA 02115, USA; 3 Medical School, University of Groningen, 9712 Groningen, The Netherlands; 4 Division of Infectious Diseases, Department of Medicine, School of Health Sciences, University of Patras, 26504 Patras, Greece; 5 Division of Infectious Diseases and Travel Medicine, Department of Medicine, Mount Auburn Hospital, Cambridge, MA 02138, USA; 6 Molecular Biology and Microbiology and Ophthalmology, Tufts Graduate School of Biomedical Sciences, Tufts University School of Medicine, Boston, MA 02111, USA

**Keywords:** tick-borne infection, climate change, expanding habitat, emerging infectious disease

## Abstract

Background: Powassan virus is an emerging neurotropic arbovirus transmitted by the tick *Ixodes scapularis.* This systematic review was conducted to aggregate data on its clinical manifestations, diagnostic findings, and complications. Methods: PubMed was searched until August 2023 using the term “Powassan”, to identify all published cases of Powassan virus infections, as per PRISMA guidelines. Results: Among the 380 abstracts identified, 45 studies describing 84 cases (70 adult, 14 pediatric) were included. Cases were reported from the USA and Canada. Complications included paralysis in 44.1% of adult and 42.6% of pediatric cases, cognitive deficits in 33.3% of adult and 25% of pediatric cases, while the mortality rate was 19.1% and 7.1% in the adult and pediatric populations, respectively. Correlation analysis revealed an association between mortality and age (r = 0.264, *p* = 0.029), development of paralysis (r = 0.252, *p* = 0.041), or respiratory distress or failure (r = 0.328, *p* = 0.006). Factors associated with persistent neurological deficits were development of ataxia (r = 0.383, *p* = 0.006), paralysis (r = 0.278, *p* = 0.048), speech disorder (r = 0.319, *p* = 0.022), and cranial nerve involvement (r = 0.322, *p* = 0.017). Other significant correlations included those between speech disorders and ataxia (r = 0.526, *p* < 0.001), and between paralysis and respiratory distress or failure (r = 0.349, *p* = 0.003). Conclusion: Powassan virus infections have significant morbidity and mortality and should be suspected in cases of encephalitis and possible tick exposure. PROSPERO registration number: CRD42023395991.

## 1. Introduction

Powassan virus is an arbovirus and a member of the Flaviviridae family. It is a single-stranded, positive-sense RNA virus, and has significant genetic similarities with another arbovirus, the deer tick virus, with a 84% nucleotide sequence identity [1]. It is also closely related to the tick-borne encephalitis virus, which is found in Asia and Europe [2]. It was originally isolated in 1958 from the brain of a child with fulminant encephalitis of unknown origin, from the town of Powassan in Ontario, Canada [3].

Powassan virus infection mainly manifests as encephalitis. However, the illness severity can vary significantly, from asymptomatic infection to the development of meningitis and encephalitis [4]. Lymphocytic infiltration of perivascular neuronal tissue can be seen on pathology, with a predilection for gray matter, including the thalamus, midbrain, and cerebellum [5]. In patients that develop meningoencephalitis, complications can include seizures, aphasia, cranial nerve palsies, and paresis [6], with approximately 10% of cases resulting in death [7].

The virus is transmitted by tick bites and has been isolated in various species, such as *Ixodes cookei* (groundhog tick), *I. scapularis* (black-legged or deer tick), *I. marxi* (squirrel tick), *I. spinipalpus*, and *Dermacentor andersoni* [8]. Cases of Powassan virus reflect the ecology of its hosts and their interaction with humans. In general, cases begin increasing in April, peaking during the summer and continuing into the fall, before decreasing during the winter [9,10]. Unlike *Borrelia burgdorferi* or *Babesia microti (*pathogens carried by *I. scapularis),* which require 48 h and 24 h of tick attachment for transmission to occur, respectively [11], Powassan virus transmission can occur 15–30 min after tick attachment, with symptoms appearing 1–4 weeks later [12].

**Figure 1:**
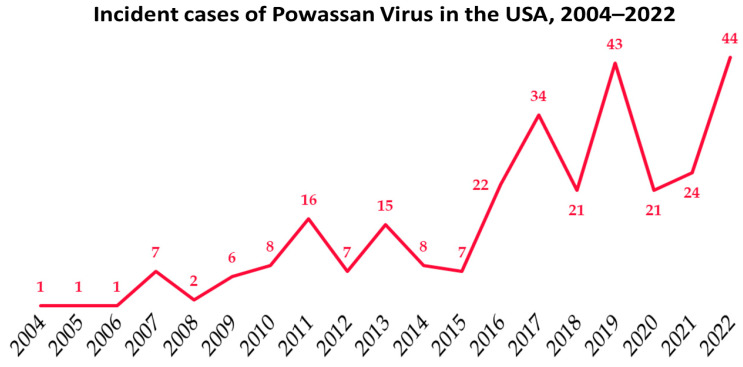
Incident cases of Powassan virus infection in the USA, as reported by the CDC [9].

Between 2004 and 2022, a total of 288 cases have been reported to the CDC, of which 267 (92.7%) pertained to neuroinvasive disease, 264 (91.7%) required hospitalization, and 36 (12.5%) resulted in death. The reported incidence of Powassan virus infection is increasing (Figure 1), with 44 incident cases noted in the USA in 2022 [9].

Given the increasing reported incidence of this devastating disease, we conducted a systematic review with the objective of identifying all published cases of Powassan virus infection, compiling aggregate data on its clinical manifestations, diagnostic findings and complications.

## 2. Materials and Methods

This systematic review was conducted according to the international Preferred Reporting Items for Systematic Reviews and Meta-Analysis (PRISMA) guidelines [13]. The study protocol has been published in the international prospective register of systematic reviews PROSPERO, (https://www.crd.york.ac.uk/prospero/display_record.php?RecordID=395991, accessed on 31 August 2023), registration number CRD42023395991.

### 2.1. Data Sources and Searches

A systematic literature review was conducted through PubMed, using the search term “Powassan [title/abstract]”. No limitations were imposed on publication date or language, with the search concluding on August 31st, 2023. Screening of article titles and abstracts was based on selection criteria as presented in Table 1. To identify further relevant publications, references from the extracted articles were also reviewed.

### 2.2. Study Selection

The selection of studies for inclusion in this review was carried out by a team of three reviewers (L.K., V.R.V., D.K.), who applied the selection criteria outlined in Table 1 to determine each study’s eligibility. Subsequently, the reviewers further assessed the relevance and quality of the studies that met the inclusion criteria.

Study design: case reports, case series, and cohort studies describing cases of Powassan virus infections.

Type of participants: adult and pediatric patients that are diagnosed with Powassan virus infection through immunoassays via the state or CDC.

Type of exposure: Powassan virus infection

Type of outcome: The primary outcome was identifying and describing the clinical manifestations, results of laboratory and imaging studies, and outcomes of patients with Powassan virus infections. Secondary outcomes were evaluating whether the presence of specific clinical manifestations and laboratory and imaging findings was associated with specific outcomes.

### 2.3. Data Extraction and Quality Assessment

The following data were extracted from full-text articles included in this review: eligibility criteria, study design, patient demographic parameters, symptoms and signs on presentation, findings of cerebrospinal fluid (CSF) and imaging studies, treatment with steroids or intravenous immunoglobulin (IVIG), development of complications, and final outcome.

The risk of bias in the included studies was independently evaluated by three authors (L.K., V.R.V., D.K.) using the Murad scale, which is a modified version of the Newcastle–Ottawa Scale (NOS) [14] used for non-randomized trials. The assessment involved the evaluation of six evidence-based criteria, which assessed for selection, representativeness of cases, ascertainment of outcomes and exposure, and adequate reporting [15]. The questions used for evaluating each study are presented in Supplementary Table S1. Discrepancies between the reviewers were resolved through group discussions until a consensus was reached. Based on the number of criteria met, a study was categorized as having low risk of bias (all 6 criteria met), moderate risk of bias (5 or 4 criteria met), or high risk of bias (3 or less criteria met) [16]. The scores assigned to each study are presented in Supplementary Table S2.

### 2.4. Data Synthesis and Analysis

Data relating to the primary and secondary endpoints are extracted and presented descriptively. Statistical analysis was performed using IBM SPSS for Windows version 28. For continuous variables, means are reported for parametric variables and medians for non-parametric variables. Statistical analysis was also conducted in order to evaluate the effect of the presence of specific manifestations, laboratory and imaging findings on mortality or long-term neurological deficits. The data were analyzed by converting them into binary variables, and correlations between these variables were identified using Pearson correlation. *p*-values were calculated using Fisher’s Exact Test (2-sided), and only *p*-values less than 0.05 were considered statistically significant.

### 2.5. Role of the Funding Source

This research did not receive any specific grant from funding agencies in the public, commercial, or not-for-profit sectors.

## 3. Results

**Figure 2:**
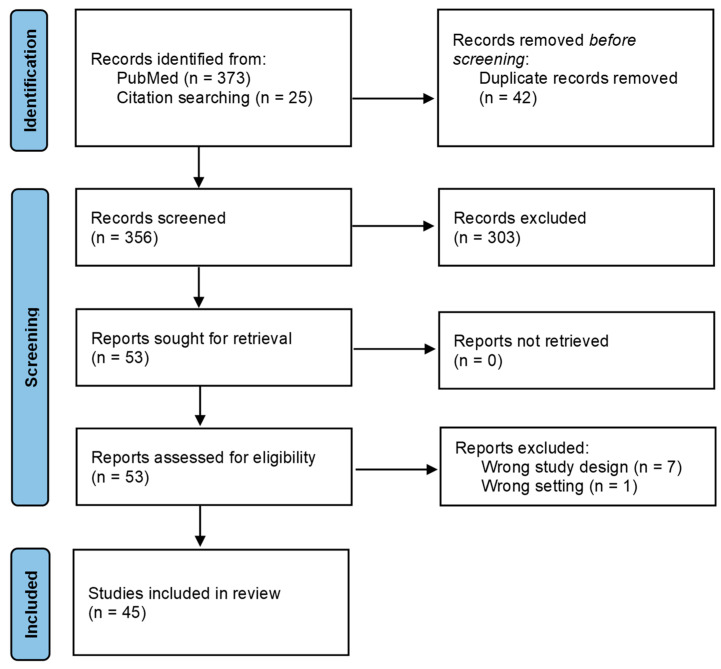
Flow chart of evaluated studies, as per PRISMA guidelines [13].

The original PubMed search retrieved 373 articles, and citation searching led to the identification of an additional 25 articles. A total of 45 studies met the inclusion criteria and were included in the review (Figure 2). The extracted data are presented in Table 2, while the quality assessment of each study is provided in Supplementary Table S2.

### 3.1. Demographics

A total of 84 cases were identified; 70 were adults and 14 were pediatric patients. Among the adult patients, 23 individuals (32.9%) were female. The mean age of the adult patients was 60.1 years. In the pediatric population, three patients (21.4%) were female, with a mean age of 4.8 years. The diagnosis was confirmed in all cases through testing of the serum samples or CSF for Powassan virus-neutralizing antibodies or genetic material. Specifically, immunoassays for Powassan virus IgM were used to identify 80 cases (95.2%)—either in serum for 61 cases (72.6%), in CSF for 38 cases (45.2%), or in both for 17 cases (20.2%). The virus was identified in the CSF through either PCR for 6 cases (7.1%) or metagenomic next-generation sequencing in 2 cases (2.4%). In total, genetic testing was the sole diagnostic method used in four cases (4.8%).

### 3.2. Geographic Distribution

**Figure 3:**
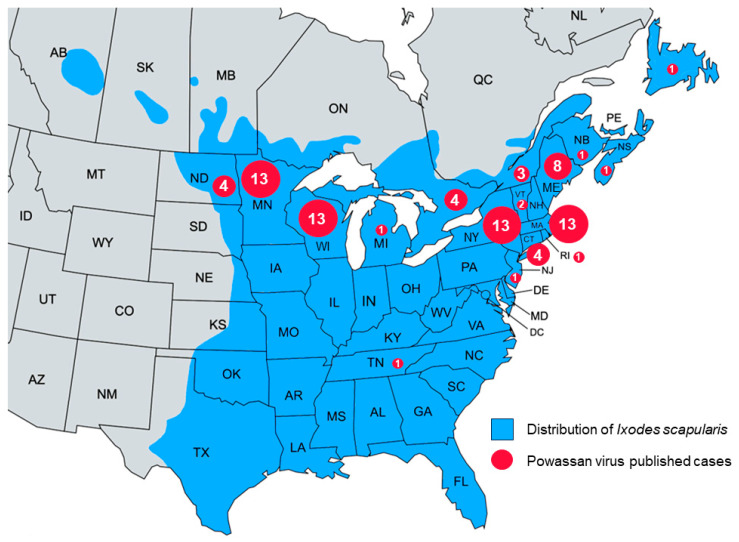
Map depicting the distribution of Powassan virus cases reported in this review along with the distribution of *Ixodes scapularis* in the USA [17] and Canada [18].

All identified cases were recorded in North America, with 75 cases (89.3%) reported from the USA and 9 cases (10.7%) from Canada. The cases were mostly distributed between northeastern United States, midwestern states bordering Canada, and Canadian states on the border with the USA. Most cases were recorded in New York, Massachusetts, Minnesota, and Wisconsin with 13 cases each. Maine followed with 8 cases; Ontario, Connecticut, and North Dakota had 4 cases each; and Quebec had 3 cases. Vermont had 2 cases, while Michigan, Tennessee, New Jersey, Rhode Island, New Brunswick, Nova Scotia, and Newfoundland each had 1 recorded case (Figure 3).

### 3.3. Data on Individual Cases

Data on individual cases, including patient presentation, CSF, and imaging findings, as well as final outcome are presented in Table 1.

**Table 2:** Data on each individual published case.

1st Author, Year	Patient Presentation	CSF Studies	Imaging Findings	Outcome
McLean, 1959 [3]	5M, right-sided headache, drowsiness, fever, twitching, tremors, nystagmus, followed by worsening mentation, neck stiffness, left-sided hemiplegia, ataxia	WBC: 150 c/mL, PMN: 60 c/mL, LY: 90 c/mL, MONO: NA, Glu: NA, Prot: NA, sPVAb +	NA	Death
Goldfield, 1973 [19]	57F, severe retro-orbital headache, hearing loss, dizziness, lethargy, delirium	WBC: 44 c/mL, PMN: 10 c/mL, LY: 35 c/mL, MO: NA, Glu: 41 mg/dL, Prot: NA, sPVAb +	NA	Persistent neurological deficit
Smith, 1974 [20]	7M, sudden-onset fever 39–40 °C, headache, three generalized tonic–clonic seizures lasting 5 min each	WBC: 460 c/mL, PMN: 438 c/mL, LY: 22 c/mL, MONO: NA, Glu: 77 mg/dL, Prot: 36 mg/dL, sPVAb 1:80	NA	Improved
	14-month M, sudden onset of URI, sore throat, fever 39 °C, a single convulsion followed by status epilepticus	WBC: 152 c/mL, PMN: 52 c/mL, LY: 100 c/mL, MONO: NA, Glu: 60 mg/dL, Prot: 138 mg/dL, sPVAb 1:1280	NA	Persistent neurological deficit, movement
	12M, fever, convulsions, somnolence, disorientation, severe headache	WBC: 77 c/mL, PMN: 16 c/mL, LY: 61 c/mL, MONO: NA, Glu: 72 mg/dL, Prot: 31 mg/dL, sPVAb 1:1280	NA	Improved
Rossier, 1974 [21]	8M, headache, malaise, anorexia, vomiting, fever, somnolence, neck stiffness, followed by stupor, seizures. Progressed to coma, right gaze deviation, left facial palsy, and bilateral pyramidal tract signs	WBC: 495 c/mL, PMN: 406 c/mL, LY: 89 c/mL, MONO: NA, Glu: 74 mg/dL, Prot: 28 mg/dL, sPVAb +	Normal reading	Improvement with physical and speech therapy
Wilson, 1979 [22]	13-month F, 4-day history of anorexia, lethargy, fever 38–39 °C, rash	WBC: NA, PMN: 175 c/mL, LY: 36 c/mL, MONO: NA, Glu: 30 mg/dL, Prot: NA, sPVAb 1:160	NA	Persistent neurological deficit, movement and cognitive
Partington, 1980 [23]	7M, 3-day history of fever, vomiting, increasing lethargy, headache	WBC: 53 c/mL, PMN: NA, LY: NA, MONO: NA, Glu: 63 mg/dL, Prot: 28 mg/dL, sPVAb 1:80	NA	Persistent neurological deficit, movement and cognitive
Embil, 1983 [24]	8M, malaise, olfactory hallucination, reduced appetite, seizures, fever	WBC: 91 c/mL, PMN: NA, LY: NA, MONO: 91 c/mL, Glu: NA, Prot: NA, sPVAb 1:320	NA	Complete recovery
Fitch, 1990 [25]	76M, 4-day history of malaise, fever, intermittent headache, vomiting	WBC: NA, PMN: NA, LY: NA, MO: NA, Glu: 107 mg/dL, Prot: NA, sPVAb 1:80	NA	Persistent neurological deficit, cognitive
Gholam, 1999 [26]	64M, headache, fever, expressive and nominal dysphasia, mild right facial weakness	WBC: 106 c/mL, PMN: 69 c/mL, LY: 34 c/mL, MO: 3 c/mL, Glu: 55 mg/dL, Prot: 175 mg/dL, sPVAb 1:160	Normal reading	Improved neurologically, died during hospitalization from a massive pulmonary embolism
Courtney, 2001 [27]	70M, fever, generalized muscle weakness, somnolence, diarrhea, anorexia	WBC: 40 c/mL, PMN: NA, LY: 35 c/mL, MO: NA, Glu: 96 mg/dL, Prot: 640 mg/dL, sPVAb 1:1640	Parietal changes consistent with microvascular ischemia or demyelinating disease	Persistent neurological deficit, movement
	53F, agitation, ataxia, bilateral lateral gaze palsy, dysarthria, loss of balance, visual disturbance, fever	WBC: 148 c/mL, PMN: 68 c/mL, LY: 59 c/mL, MO: NA, Glu: NA, Prot: NA, sPVAb 1:640	Bilateral temporal lobe microvascular ischemia or demyelinating disease	Residual deficits, persistent neurological deficit
	25M, fever, headache, vomiting, somnolence, confusion, bilateral hand twitching, muscle weakness, pronounced lip smacking	WBC: 920 c/mL, PMN: NA, LY: 681 c/mL, MO: NA, Glu: 77 mg/dL, Prot: 80 mg/dL, cPVAb 1:80	NA	Improved
	66M, somnolence, severe headache, increasing confusion, progressive bilateral leg weakness, slow speech, memory loss, wide-based gait	WBC: 54 c/mL, PMN: NA, LY: 51 c/mL, MO: NA, Glu: 67 mg/dL, Prot: 640 mg/dL, sPVAb 1:640	NA	Persistent neurological deficit, cognitive
Lessell, 2003 [28]	53F, nausea, vomiting, diarrhea, dizziness, diplopia, ataxia, followed by arm weakness, urinary retention, fever, delirium, ophthalmoplegia	WBC: 148 c/mL, PMN: 68 c/mL, LY: 59 c/mL, MO: 21 c/mL, Glu: NA, Prot: 640 mg/dL, sPVAb 1:640	Hyperintensity in the white matter of each temporal lobe	Persistent neurological deficit
Hinten, 2008 [7]	70M, Somnolence, AMS, generalized muscle weakness progressing to left-sided hemiplegia, encephalopathy, renal insufficiency, anemia.	WBC: 40 c/mL, PMN: 5 c/mL, LY: 35 c/mL, MO: 0 c/mL, Glu: NA, Prot: 96 mg/dL, cPVAb 1:640.	Old infarct in right parietal lobe, bilateral parietal lobe abnormalities suggestive of microvascular ischemia or demyelinating disease	Dense left-sided hemiplegia at the time of discharge
	53F, ataxia, bilateral lateral gaze palsy, dysarthria, AMS, generalized muscle weakness, complete ophthalmoplegia	WBC: 148 c/mL, PMN: 68 c/mL, LY: 59 c/mL, MO: 0 c/mL, Glu: NA, Prot: NA, sPVAb 1:640.	Bilateral temporal lobe abnormalities consistent with microvascular ischemia or demyelinating disease	Ophthalmoplegia
	25M, fever 38.5 °C, headache, vomiting, somnolence, confusion, inability to walk, bilateral hand twitching, bilateral upper extremity weakness, pronounced lip-smacking	WBC: 920 c/mL, PMN: 239 c/mL, LY: 681 c/mL, MO: 0 c/mL, Glu: 77 mg/dL, Prot: 80 mg/dL, cPVAb 1:80	NA	Persistent neurological deficit, movement
	74F, headache, fever 40.5 °C, myalgias, confusion, progressive weakness with inability to speak or walk, combative, tremors	WBC: 28 c/mL, PMN: 5 c/mL, LY: 23 c/mL, MO: 0 c/mL, Glu: 34 mg/dL, Prot: 8 mg/dL, cPVAb 1:8	NA	Persistent neurological deficit, movement
	66M, somnolence, severe headache, increasing confusion, slow speech, short-term memory loss, bilateral leg weakness, wide-based gait	WBC: 54 c/mL, PMN: 3 c/mL, LY: 51 c/mL, MO: 0 c/mL, Glu: 67 mg/dL, Prot: 640 mg/dL, sPVAb 1:640	NA	Persistent neurological deficit, cognitive
	69M, abdominal pain, vomiting, fever 38.7 °C, chills, lethargy	WBC: 60 c/mL, PMN: NA, LY: NA, MO: NA, Glu: NA, Prot: 60 mg/dL, cPVAb 1:640	NA	NA
	60F, fever 39.2 °C, diplopia, acute onset of proximal muscle weakness in all extremities (upper > lower), paralysis, respiratory failure requiring mechanical ventilation.	WBC: 198 c/mL, PMN: NA, LY: NA, MO: NA, Glu: 61 mg/dL, Prot: 16 mg/dL, cPVAb 1:16	NA	Persistent B/L upper extremity weakness requiring total assistance with feeding and dressing
	83M, fever, headache, AMS, stiff neck, generalized muscle weakness	WBC: 80 c/mL, PMN: 42 c/mL, LY: 38 c/mL, MO: 0 c/mL, Glu: 61 mg/dL, Prot: 320 mg/dL, sPVAb 1:320	NA	Improvement in symptoms
	91F, fever, headache, AMS, stiff neck, muscle pain, generalized muscle weakness	WBC: 28 c/mL, PMN: 3 c/mL, LY: 25 c/mL, MO: 0 c/mL, Glu: 64 mg/dL, Prot: 960 mg/dL, sPVAb 1:960	Cerebral atrophy	Improvement in symptoms
Tavakoli, 2009 [29]	62M, fatigue, fever, bilateral maculopapular palmar rash, diplopia, dysarthria, weakness of right arm and leg	WBC: 891 c/mL, PMN: 9 c/mL, LY: 829 c/mL, MO: NA, Glu: 47 mg/dL, Prot: 192 mg/dL, cPVAb +, cPCR +	Hyperintensities in superior cerebellum, left pons, and bilateral basal ganglia, restricted diffusion in the superior cerebellum	Death
Trépanier, 2010 [30]	61M, 3 days of fever, AMS, disorientated with incoherent speech, dizziness, headache	WBC: 65 c/mL, PMN: 9 c/mL, LY: NA, MO: NA, Glu: NA, Prot: NA, sPVAb 1:40.	FLAIR/T2 hyperintensity at the junction between the left internal capsule and the caudate nucleus and of the junction between putamen and external capsule	Improved
Hicar, 2011 [8]	9F, 3 days of headache, abdominal pain, emesis, fever to 39.4 °C, nuchal rigidity	WBC: 68 c/mL, PMN: 44 c/mL, LY: 17 c/mL, MONO: 10 c/mL, Glu: 63 mg/dL, Prot: 52 mg/dL, sPVAb +	T2 hyperintensities in the white matter, left putamen, and caudate nucleus	Persistent neurological deficit, movement, and cognitive
Raval, 2012 [31]	18M, severe headache for 2 days, 1 episode of seizure	WBC: 237 c/mL, PMN: 218 c/mL, LY: 19 c/mL, MO: NA, Glu: 66 mg/dL, Prot: 53 mg/dL, sPVAb +	Normal reading	Improved
	60M, fevers, headaches, dizziness	WBC: 12 c/mL, PMN: NA, LY: 12 c/mL, MO: NA, Glu: 166 mg/dL, Prot: 80 mg/dL, cPVAb 1:640	NA	Improved
	61M, progressive headaches, body aches, high-grade fever, AMS	WBC: 3 c/mL, PMN: NA, LY: NA, MO: NA, Glu: 57 mg/dL, Prot: 46 mg/dL, cPCR +	Normal reading	Improved
	69M, progressive weakness, headaches, fevers	NA. Diagnosis through cPCR +	NA	Persistent neurological deficit, movement
Choi, 2012 [32]	43M, fever, chills, arthralgia, myalgia, rash, diarrhea, sudden-onset left-sided weakness, progressive AMS	WBC: 60 c/mL, PMN: NA, LY: NA, MO: NA, Glu: NA, Prot: NA, sPVAb +	Right thalamic lesion with restricted diffusion	Persistent neurological deficit, movement
Birge, 2012 [5]	67F, 3-day history of dizziness, fever 39.4 °C, chills, malaise, nausea, confusion, dysarthria, mild neck tenderness	WBC: 80 c/mL, PMN: 71 c/mL, LY: 9 c/mL, MO: NA, Glu: NA, Prot: 64 mg/dL, sPVAb +, cPVAb +	Nonspecific inflammatory changes within the thalamus, midbrain, and cerebellum. Mass effect of structures at the foramen magnum, acute hydrocephalus	Death
Sung, 2013 [33]	22M, influenza-like illness, recurred after few weeks with fever, eye pain, lateral gaze palsy, ataxia, dysarthria, stomach pain, neck stiffness	WBC: 212 c/mL, PMN: 11 c/mL, LY: 201 c/mL, MO: NA, Glu: 60 mg/dL, Prot: 55 mg/dL, cPVAb 1:320	Bilateral caudate and basal ganglia hyperintensities consistent with encephalitis	Persistent dysarthria, mild tremors
	34M, rash on trunk followed by headache, fever, chills, bilateral ankle pain, progressed to bilateral proximal leg weakness, confusion, diplopia	WBC: 145 c/mL, PMN: NA, LY: NA, MO: NA, Glu: 39 mg/dL, Prot: 142 mg/dL, cPVAb 1:320	Hyperintensities in bilateral temporal lobes	Residual leg weakness
Piantadosi, 2016 [34]	82M, sudden onset of dizziness followed by fever, nausea, vomiting.	WBC: 169 c/mL, PMN: NA, LY: 140 c/mL, MO: 0 c/mL, Glu: 45 mg/dL, Prot: 230 mg/dL, cPVAb 1:8	Diffuse T2 signal intensity in cerebellum and vermis	Death
	74M, 2 weeks of upper respiratory symptoms, followed by fever, right eye pain, visual blurring	WBC: 108 c/mL, PMN: 33 c/mL, LY: 55 c/mL, MO: NA, Glu: 76 mg/dL, Prot: 201 mg/dL, cPVAb 1:512	T2 and FLAIR hyperintensities throughout the brainstem and basal ganglia bilaterally, extending to the right anterior frontal subcortical white matter	Persistent neurological deficit, movement, and cognitive
	21M, 3 days of vomiting, confusion, fever, rash	WBC: 156 c/mL, PMN: 47 c/mL, LY: 80 c/mL, MO: NA, Glu: 85 mg/dL, Prot: 320 mg/dL, sPVAb 1:320	T2/FLAIR hyperintensities in basal ganglia (insula, caudate heads, putamen) and thalami bilaterally and diffusion restriction throughout the cortex.	Improved
	67M, 4 days of confusion, encephalopathy, vomiting, diarrhea, fever, faint maculopapular rash over the chest, upper back.	WBC: 557 c/mL, PMN: 11 c/mL, LY: 418 c/mL, MO: NA, Glu: 49 mg/dL, Prot: 89 mg/dL, sPVAb 1:160	T2/FLAIR hyperintensities in the caudate heads, putamina, and thalami	Improved
	65F, 7 days of fever, confusion, slow speech, headache, vomiting	WBC: 220 c/mL, PMN: 31 c/mL, LY: 161 c/mL, MO: NA, Glu: 44 mg/dL, Prot: 84 mg/dL, sPVAb 1:160	T2/FLAIR hyperintensity was visible involving the basal ganglia bilaterally	Improved
	52M, 2 days of fever, myalgias	WBC: 420 c/mL, PMN: 8 c/mL, LY: 336 c/mL, MO: NA, Glu: 53 mg/dL, Prot: 113 mg/dL, sPVAb 1:160	T2/ FLAIR hyperintensity in bilateral basal ganglia and thalami, diffusion restriction in dorsal mid-brain	Persistent neurological deficit, movement, and cognitive
	49M, 4 days of fever, headache	WBC: 146 c/mL, PMN: NA, LY: 136 c/mL, MO: NA, Glu: 54 mg/dL, Prot: 107 mg/dL, sPVAb +	Asymmetric T2/FLAIR hyperintensities in the basal ganglia and thalami	Death
	44M, 3 days of headache, fatigue, diplopia, diffuse rash over trunk and extremities	WBC: 720 c/mL, PMN: 288 c/mL, LY: 338 c/mL, MO: NA, Glu: 91 mg/dL, Prot: 58 mg/dL, sPVAb 1:10240 (reported concern for cross reactivity with other arboviruses)	Normal reading	Improved
Cavanaugh, 2017 [35]	72F, erythema migrans on left scapula, myalgias, chills, fever, headache, AMS, hypotension, oliguria	WBC: 119 c/mL, PMN: 115 c/mL, LY: NA, MO: NA, Glu: 79 mg/dL, Prot: 42 mg/dL, sPVAb 1:2560	T2 hyperintensities in cerebellar cortex and vermis, pons, midbrain, ventrolateral thalami, and dentate nuclei	Death
Tutolo, 2017 [36]	5-month M, fever, vomiting, right-sided facial twitching that progressed to seizures	WBC: 125 c/mL, PMN: NA, LY: 101 c/mL, MONO: NA, Glu: NA, Prot: 32 mg/dL, cPVAb 1:32	Restricted diffusion involving the basal ganglia, rostral thalami, and left pulvinar, consistent with encephalitis	Persistent neurological deficit
Mittal, 2017 [37]	35F, headache, vomiting, confusion, fever 38.4 °C, severe hypertension (240/140 mm Hg)	WBC: 343 c/mL, PMN: 7 c/mL, LY: NA, MO: 336 c/mL, Glu: 47 mg/dL, Prot: 76 mg/dL, cPVAb 1:32.	T2 white matter hyperintensities in the cerebral hemispheres and posterior fossa bilaterally	Persistent neurological deficit, movement, and cognitive
Sanderson, 2018 [38]	68F, right abducens nerve palsy, mild left-sided pyramidal weakness, erythematous mark on right shoulder	WBC: 31 c/mL, PMN: NA, LY: 28 c/mL, MO: NA, Glu: 54 mg/dL, Prot: 105 mg/dL, sPVAb 1:5120.	T2 and FLAIR hyperintensities in supratentorial white matter	Persistent neurological deficit, movement
Solomon, 2018 [39]	60M, 1 week of testicular pain, fever, testicular ultrasonography demonstrated orchiepididymitis. Later developed meningismus, bilateral upper extremity dysmetria, dysarthria, gait instability	WBC: 10 c/mL, PMN: 4 c/mL, LY: 6 c/mL, MO: NA, Glu: 62 mg/dL, Prot: 83 mg/dL, cPCR +, sPCR +	Diffuse cerebellar edema, obstructive hydrocephalus, diffuse leptomeningeal enhancement, and periventricular, thalamo-mesencephalic, and basal ganglia T2 hyperintensities	Death
Patel, 2018 [40]	81F, fever 41.1 C, somnolence, neck stiffness, diffuse motor weakness, increased tone and cogwheel rigidity, nonconvulsive status epilepticus.	WBC: 197 c/mL, PMN: 2 c/mL, LY: 160 c/mL, MO: 18 c/mL, Glu: 60 mg/dL, Prot: 121 mg/dL, sPVAb 1:2660	T2/FLAIR hyperintensities in superficial and deep white matter and corpus callosum	Improved
Picheca, 2019 [41]	62M, nausea, vomiting, abdominal pain, diplopia, ataxia, dysarthria, respiratory distress, weakness progressing to flaccid paralysis (upper > lower) with preserved sensation	WBC: 159 c/mL, PMN: 67 c/mL, LY: 68 c/mL, MO: NA, Glu: 79 mg/dL, Prot: 160 mg/dL, sPVAb 1:160	Infratentorial and supratentorial leptomeningeal enhancement. T2 hyperintensity signal involving the anterior horns from C3 to C6	NA
Khan, 2019 [42]	87M, 2 weeks of worsening fatigue, malaise, intermittent lightheadedness, mild abdominal pain followed by fever 39.4 °C, rigors, vomiting, drowsiness. Co-infected with *Babesia microti* and *Borrelia burgdorferi.*	WBC: 20000 c/mL, PMN: NA, LY: 19200 c/mL, MO: 800 c/mL, Glu: 60 mg/dL, Prot: 48 mg/dL, sPVAb +	Normal reading	Persistent neurological deficit, movement, and cognitive
Allgaier, 2019 [43]	55M, headache, nausea, vomiting followed by confusion, memory loss	WBC: 88 c/mL, PMN: 4 c/mL, LY: 74 c/mL, MO: 11 c/mL, Glu: 64 mg/dL, Prot: NA, sPVAb +, cPVAb +	T2 hyperintensities in bilateral caudate, putamen, and hippocampus, findings of inflammatory encephalitis. Enhancement of hippocampus in DWI	Short-term memory deficit, resolved within 5 months
Colman, 2020 [44]	56M, worsening headache, decreased appetite, vomiting, trouble dressing, acute confusion, new-onset blurred vision.	WBC: 218 c/mL, PMN: NA, LY: 201 c/mL, MO: NA, Glu: NA, Prot: NA, sPVAb +	FLAIR hyperintensity about the cerebral convexities and cerebellar folia, leptomeningeal enhancement	Improved
Koester, 2020 [45]	8F, 1-day history of headache, photophobia, fever to 38.3 °C, lethargy, poor oral intake, diffuse abdominal pain, vomiting	WBC: 88 c/mL, PMN: 20 c/mL, LY: 58 c/mL, MONO: 10 c/mL, Glu: 66 mg/dL, Prot: 35 mg/dL, cPVAb 1:40.	Normal reading	Improved
Yu, 2020 [46]	88M, fever, AMS, dysarthria, falls, left arm weakness, hypoxic respiratory failure due to aspiration	WBC: 33 c/mL, PMN: NA, LY: 30 c/mL, MO: NA, Glu: NA, Prot: NA, cPCR +	Normal reading	Death
Feder, 2021 [47]	5-month M, 2 days of fever (39.4 °C), vomiting, right-sided facial twitching progressing to seizures. Symptom onset 2 weeks after tick was removed from infant’s head.	WBC: 125 c/mL, PMN: 24 c/mL, LY: 101 c/mL, MONO: NA, Glu: 57 mg/dL, Prot: 55 mg/dL, cPVAb 1:32.	Edema of the basal ganglia, rostral thalami, and left pulvinar, consistent with encephalitis	Speech delay
	2-month M, Fever 38.9° C, listlessness for 1 day, followed by left-sided focal seizures	WBC: 215 c/mL, PMN: 22 c/mL, LY: 112 c/mL, MONO: 82 c/mL, Glu: 56 mg/dL, Prot: 75 mg/dL, sPVAb +, cPVAb +	Patchy edema of the thalami, right parietal lobe, and right mid brain	Paresis of the left upper extremity
Pach, 2021 [48]	62M, fevers, night sweats, headaches, fatigue, AMS, progressed to profound expressive aphasia, ataxia, unable to protect airway.	WBC: 59 c/mL, PMN: 2 c/mL, LY: 52 c/mL, MO: 5 c/mL, Glu: 80 mg/dL, Prot: 99 mg/dL, cPVAb 1:320	Mild generalized cerebral and cerebellar atrophy	Persistent ataxia and expressive aphasia with spasticity in upper limbs requiring baclofen pump, wheelchair-bound
Dumic, 2021 [49]	42M, 2 days of diplopia due to left abducens nerve palsy, dysarthria, headache, fever 38.5 °C, maculopapular rash torso	WBC: 193 c/mL, PMN: NA, LY: 154 c/mL, MO: NA, Glu: NA, Prot: NA, cPVAb 1:320	Normal reading	Complete recovery
Dumic, 2021 [50]	76M, confusion followed by abrupt onset of fever the next day. Co-infected with *Borrelia burgdorferi.*	WBC: 68 c/mL, PMN: NA, LY: 37 c/mL, MO: NA, Glu: NA, Prot: NA, cPVAb 1:8	Normal reading	Persistent neurological deficit, movement, and cognitive
Taylor, 2021 [51]	30F, severe headache, fever, weakness, myalgias, chills, confusion, photophobia, nausea, diarrhea, 24 days after undergoing kidney transplantation	WBC: 5 c/mL, PMN: NA, LY: NA, MO: NA, Glu: 55 mg/dL, Prot: 320 mg/dL, sPVAb 1:320	Abnormal T2-FLAIR signal in the cerebellum	Persistent neurological deficit, cognitive
Kroopnick, 2021 [52]	82F, 2 days of worsening back pain, headache, vomiting, followed by acute agitation and confusion. Co-infected with *Anaplasma phagocytophilum.*	NA, cPVAb +	T2/FLAIR hyperintensities dorsal midbrain, pons, and superior cerebellar peduncles	Death
Nord, 2021 [53]	51M, acute AMS, fever 40 °C	NA, cPVAb +	NA	Improved
Bazer, 2022 [54]	62M, acute onset AMS, dysarthria, left-sided facial droop, followed by recurrent strokes	WBC: 370 c/mL, PMN: 296 c/mL, LY: 74 c/mL, MO: NA, Glu: 59 mg/dL, Prot: 152 mg/dL, cPVAb +	Acute infarct involving cerebellum, left basal ganglia, and splenium of corpus callosum, left putamen infarct	Global aphasia at the time of discharge
Johnson, 2022 [55]	68M, headache, recurrent fever, rapidly progressive weakness	WBC: 274 c/mL, PMN: 38 c/mL, LY: 236 c/mL, MO: NA, Glu: 107 mg/dL, Prot: 84 mg/dL, cPVAb +	Cerebellar cerebritis without hydrocephalus	Found to have diffuse large B-cell lymphoma, transitioned to comfort care
	75M, headache, encephalopathy, inability to follow commands, rigidity with saccades, vertical upgaze restriction	WBC: 76 c/mL, PMN: 8 c/mL, LY: 68 c/mL, MO: NA, Glu: 64 mg/dL, Prot: 34 mg/dL, CSF mNGS +	Cerebral cerebritis and obstructive hydrocephalus	No neurological improvement, comfort care
Kakoullis, 2022 [56]	75F, hypersomnia, apraxia, AMS, short-term memory loss, encephalitis	WBC: 69 c/mL, PMN: 2 c/mL, LY: 61 c/mL, MO: 6 c/mL, Glu: 49 mg/dL, Prot: 60 mg/dL, cPVAb +	Bilaterally increased T2 signal in posterior parietal lobes, cerebellum, and basal ganglia	Persistent neurological deficit, cognitive
Mendoza, 2023 [57]	75M, encephalopathy, anarthria, quadriparesis, vertical gaze restriction	WBC: 6 c/mL, PMN: 3 c/mL, LY: 2 c/mL, MO: NA, Glu: NA, Prot: NA, CSF mNGS +	Cerebellitis with obstructive hydrocephalus	Death
	74F, left-sided weakness, dysarthria, tremor, truncal ataxia	WBC: 121 c/mL, PMN: 17 c/mL, LY: 87 c/mL, MO: NA, Glu: NA, Prot: NA, sPVAb +, sPCR +	Symmetric T2 hyperintensities in bilateral thalami	NA
	34F, headache, fever, nausea, AMS	WBC: 257 c/mL, PMN: 8 c/mL, LY: 242 c/mL, MO: NA, Glu: NA, Prot: NA, sPVAb 1:640	NA	Cognitive impairment and headaches
	2-month M, seizures	WBC: 480 c/mL, PMN: 230 c/mL, LY: 62 c/mL, MO: NA, Glu: NA, Prot: NA, sPVAb 1:640	Normal	Recovery
	68M, rapidly progressive quadriparesis, encephalopathy, multiple cranial neuropathies (left 3rd and 4th, bilateral 6th, and bilateral optic perineuritis)	WBC: 274 c/mL, PMN: 0 c/mL, LY: 236 c/mL, MO: NA, Glu: NA, Prot: NA, cPVAb +	Cerebellitis and ventral cervical cord enhancement	Death
	50F, headache, fever, neck pain	WBC: 40 c/mL, PMN: 16 c/mL, LY: 16 c/mL, MO: NA, Glu: NA, Prot: NA, sPVAb 1:640	Normal	NA
	76M, headache, fever, neck pain	WBC: 55 c/mL, PMN: 32 c/mL, LY: 9 c/mL, MO: NA, Glu: NA, Prot: NA, sPVAb 1:2560	Normal	Cognitive impairment
	76M, encephalopathy, multifocal myoclonus	WBC: 85 c/mL, PMN: 18 c/mL, LY: 43 c/mL, MO: NA, Glu: NA, Prot: NA, sPVAb 1:640	Normal	Cognitive impairment and gait disturbance
	49M, left 6th nerve palsy, dysarthria, upper-extremity dysmetria	WBC: 193 c/mL, PMN: 15 c/mL, LY: 176 c/mL, MO: NA, Glu: NA, Prot: NA, sPVAb 1:40	Cerebellar and occipital lobe leptomeningeal enhancement	Gait disturbance
	58M, headaches, dysarthria, appendicular and truncal ataxia	WBC: 66 c/mL, PMN: 5 c/mL, LY: 57 c/mL, MO: NA, Glu: NA, Prot: NA, sPVAb 1:160	Leptomeningeal enhancement at cerebellar folia and cerebral hemispheres	Gait disturbance
	74M, truncal > appendicular ataxia, encephalopathy, anarthria	WBC: 5 c/mL, PMN: 0 c/mL, LY: 3 c/mL, MO: NA, Glu: NA, Prot: NA, cPVAb <1:10	Diffuse sulcal hyperintensities on post-gadolinium FLAIR sequence	Persistent ataxia and dysarthria
	33F, headache, encephalopathy, ataxia and left 6th nerve palsy	WBC: 585 c/mL, PMN: 6 c/mL, LY: 579 c/mL, MO: NA, Glu: NA, Prot: NA, cPVAb 1:32	Midbrain, pons, and cerebellar FLAIR hyperintensities	Parkinsonism
	71F, headache, fever, meningismus, encephalopathy	WBC: 43 c/mL, PMN: 1 c/mL, LY: 39 c/mL, MO: NA, Glu: NA, Prot: NA, sPVAb 1:1280	Normal	Gait disturbance, headaches
	78F, fever, headache, encephalopathy	WBC: NA c/mL, PMN: NA c/mL, LY: NA c/mL, MO: NA, Glu: NA, Prot: NA, sPVAb 1:640	Normal	Recovery
	44M, fever, headache, meningismus, truncal ataxia, tremulousness, opsoclonus	WBC: 860 c/mL, PMN: NA c/mL, LY: 705 c/mL, MO: NA, Glu: NA, Prot: NA, sPVAb 1:640	Normal	Recovery
	56F, encephalopathy, truncal ataxia, quadriparesis, dysarthria, left 6th nerve palsy	WBC: 153 c/mL, PMN: 53 c/mL, LY: 58 c/mL, MO: NA, Glu: NA, Prot: NA, cPVAb +	Leptomeningeal enhancement and left basal ganglia FLAIR hyperintensities	Death

Abbreviations: M, male; F, female; WBC, white blood cell count; PMN, polymorphonuclear; LY, lymphocytes; MO, monocytes; Glu, glucose; Prot, protein; cPVAb, cerebrospinal fluid Powassan virus-neutralizing antibody titers; sPVAb, serum Powassan virus-neutralizing antibody titers; +, positive immunoassay, titer not reported; sPCR, serum polymerase chain reaction; cPCR, CSF polymerase chain reaction; NA, not available; DWI, diffusion-weighted imaging; mNGS, metagenomic next-generation sequencing.

### 3.4. Presentation

Findings on clinical manifestations are outlined in Table 3. In the adult patients, fever was the most common symptom, reported by 77.1% of patients, followed by headache, affecting 54.3% of patients. Other common symptoms included confusion (42.9%), speech disorders (32.9%), weakness and fatigue (30%), nausea and vomiting (24.3%), and visual symptoms (27.1%). Less frequently reported were hypersomnia, somnolence, and lethargy (11.4%); myalgias and arthralgias (8.6%), memory loss (5.7%), and seizures (1.4%).

Signs in the adult population included altered mental status (50% of cases), paralysis or paresis (25.7%), cranial nerve involvement (25.7%), and ataxia (24.3%). Other signs included rash (14.3%), meningeal signs (12.9%), and tremors (10%).

The final diagnosis was encephalitis in 63 patients and meningoencephalitis in 16 patients; 5 patients had no final diagnosis reported. Furthermore, four patients had a concurrent tickborne infection.

In the pediatric population, fever was also the most commonly reported symptom, found in all 14 patients (100%). Seizures were the second-most frequently observed symptom (64.3%), followed by headache (50%). Hypersomnia, somnolence, lethargy, nausea, and vomiting were each reported in 42.9%. Weakness and fatigue were noted in 14.34% of patients.

Altered mental status was observed in 28.6% of pediatric cases, whereas meningeal signs, tremors, and twitching each occurred in 21.4%. Paralysis or paresis was noted in 14.3% of patients. A rash was present in 14.3% of patients, while ataxia and cranial nerve involvement were each present in 7.1% of cases. No cases of confusion, memory loss, myalgias, arthralgias, respiratory distress or failure, speech disorders, or visual symptoms were observed in the pediatric population.

The final diagnoses were encephalitis (10) and meningoencephalitis (4). No concurrent tickborne infections were noted in the pediatric population.

### 3.5. CSF Studies

Lumbar puncture was performed in 79 (94%) of patients, with findings summarized in Table 4. Among adult patients, 4 did not undergo lumbar puncture, while data were not available for 1 case. The median white blood cell (WBC) count was 119/mL, predominantly lymphocytic, with a median lymphocyte count of 59.2/mL, a median monocyte count of 1.6, and a median neutrophil count of 10.9/mL. The mean glucose level was 64.9 mg/dL, and the mean protein level was 88.2 mg/dL. Opening pressures were reported in three subjects, with a median value of 180 mm H_2_O. Powassan virus-neutralizing antibody titers were reported in 45 cases, with a median titer of 1:320.

All 14 pediatric patients underwent lumbar puncture. The median WBC count was 137.5/mL, also with a lymphocytic predominance, consisting of a median lymphocyte count of 75.5/mL, a median monocyte count of 46, and a median neutrophil count of 48.1/mL. The mean glucose level was 65.3 mg/dL and the median protein level was 35.5 mg/dL. Data on opening pressures were available for four pediatric cases, with a median value of 140 mm of H_2_O. Powassan virus-neutralizing antibody titers were measured in 10 pediatric cases, with a median titer of 1:120.

### 3.6. Imaging Studies

Brain MRI results were available for 62 cases, specifically for 56 adults and 6 pediatric patients. Findings are summarized in Table 5.

Among the adults, the most commonly affected location was the basal ganglia, observed in 32.1% of cases. This was followed by cerebral involvement (26.8%) and cerebellar involvement (21.4%). The thalamus was affected in 14.3% of cases, while the midbrain showed involvement in 8.9% of cases. Additionally, 8.9% of cases presented with leptomeningeal enhancement on MRI while 21.4% of cases showed a normal MRI result.

Among the pediatric patients, two had both basal ganglia lesions and thalamic lesions, one had basal ganglia lesions and cerebral lesions, and one had thalamic lesions and midbrain lesions, while two patients had a normal MRI result.

### 3.7. Treatment

The identified treatment modalities were the administration of corticosteroids and/or IVIG. Six adult patients received only corticosteroids, four received only IVIG, while another four received both treatments. An improvement was noted in 3 of the patients receiving only corticosteroids and in 3 of the patients receiving both steroids and IVIG.

Among the pediatric patients, two received corticosteroids, and both improved after treatment. No pediatric patients received IVIG.

### 3.8. Outcomes

Outcome data are summarized in Table 6. Among adult patients, data on mortality were available for all but two patients, with 13/68 (19.1%) patients expiring due to the disease. Regarding complications, 30/68 (44.1%) patients developed paralysis, 10/67 (14.9%) developed tremors, and 17/51 (33.3%) developed cognitive defects. The neurological deficits persisted in 32/51 (62.7%) patients for whom follow-up data were available, including paralysis in 14/18 (77.8%), tremors in 5/8 (62.5%), and cognitive defects in 14/17 (82.3%).

Among the pediatric patients, death occurred in 1/14 (7.1%) patients, paralysis was noted in 6/14 (42.6%), tremors were noted in 2/14 (14.3%), and cognitive defects were noted in 3/12 (25%). Persistent neurological deficits were noted for 6/12 (50%) of patients for whom follow-up data were available: paralysis in 3/4 (75%) and cognitive defects in 3/3 (100%). Follow-up data were available for only one of the patients who developed tremor, in whom the tremors were not noted on follow-up.

### 3.9. Correlation Analysis

Pearson correlation analysis was conducted only in the adult population due to the small sample size in the pediatric population, which limited the feasibility of such an analysis. Factors significantly correlated with mortality were age (r = 0.264, *p* = 0.029), development of paralysis (r = 0.252, *p* = 0.041), or respiratory distress or failure (r = 0.328, *p* = 0.006). Factors associated with persistent neurological deficits were development of ataxia (r = 0.383, *p* = 0.006), paralysis (r = 0.278, *p* = 0.048), speech disorder (r = 0.319, *p* = 0.022), and cranial nerve involvement (r = 0.322, *p* = 0.017). Other significant correlations included those between speech disorders and ataxia (r = 0.526, *p* < 0.001), and between paralysis and respiratory distress or failure (r = 0.349, *p* = 0.003).

## 4. Discussion

This systematic review examines Powassan virus infections, an emerging arbovirus increasingly identified as a causative agent of encephalitis in the USA [9]. The review provides a comprehensive description of reported cases, including the prevalence of symptoms, signs, CSF, and imaging findings in these patients, as well as data on outcomes and prognostic factors.

Adult patients appeared to present with typical findings of viral encephalitis, with fever, headache, fatigue, and encephalopathy being among the most common findings. Some notable findings were the presence of speech disorders, visual symptoms, paresis, ataxia, and cranial nerve involvement. These findings may serve as clues for suspecting this pathogen, distinguishing it from other causes of encephalitis, such as HSV [58]. Furthermore, imaging studies suggest a predilection for the basal ganglia, which has been associated with Flaviviridae infections [59].

Powassan virus infections were associated with significant morbidity and mortality. Among 288 cases reported to the CDC between 2004 and 2022, 36 (12.5%) had a fatal outcome [9]. That mortality rate is lower than the rate calculated from the cases included in this review (14/78, 17.9%), suggesting the presence of publication bias. Furthermore, neurological deficits occurred at a significant rate, both cognitive and movement-related, which became persistent and long-term in the majority of adult cases. Correlation analysis identified age and complications such as paralysis and respiratory distress as associated with mortality, while the development of ataxia, paralysis or paresis, cranial nerve involvement, or speech disorders was associated with the development of long-term neurological deficits.

Notably, the pediatric patients frequently experienced seizures, which were the second-most commonly reported symptom. It is notable that the prevalence of this symptom was only 1.4% in the adult population, compared to 64.3% in the pediatric population. Imaging studies were conducted less often, but when performed, they also identified the basal ganglia as a commonly affected site. The prognosis was also unfavorable in this group, as long-term neurological deficits were common and persistent.

Identification of exposure to ticks can play a crucial role in making the diagnosis. However, this task may be unreliable, as even in cases of Lyme disease, which requires prolonged tick attachment for transmission to occur, patients rarely recall tick bites [60]. Consequently, clinicians should consider other indicators, such as the presence in or travel to an endemic area, as well as the engagement in activities that increase the likelihood of exposure to ticks, such as hiking, camping, gardening, or hunting [61]. Such exposures should raise the clinician’s suspicion regarding the possibility of Powassan virus as the underlying cause of the patient’s condition.

Furthermore, the geographic distribution of cases of Powassan virus infections appears to be expanding. From the original case in Ontario [3], cases have been reported as far west as North Dakota [31] and as far south as Tennessee [8]. This may be due to increased awareness and increased testing and diagnosis. It may also be due to the expanding habitat of its host. Indeed, studies evaluating the impact of climate change on *I. scapularis* have shown that there is a progressive expansion of its range, estimated to be approximately 46 km/year. This process is expected to continue, especially northward, as more areas become warmer and more suitable habitats for the tick [62]. Furthermore, climate change is leading to warmer winters, which may translate to increased tick survival and possibly more infections [63].

There are limitations to this study. First and foremost, the information included in each case was dependent on what was reported by each study, and in several cases, the data were incomplete. Furthermore, the mortality rate calculated in this systematic review exceeds that reported by the CDC, suggesting that there is publication bias favoring the more severe cases. Information on co-morbidities, which may affect mortality and morbidity, was not extracted. Therefore, there is a significant selection bias that may increase the prevalence of more severe symptoms, signs, complications, and long-term sequalae including mortality, which may have been overestimated. In addition, although this study did not encompass reports of deer tick virus, it is noteworthy that the term “Powassan virus” is occasionally used as an umbrella term encompassing both Powassan virus and deer tick virus; consequently, the authors lack the means to ascertain whether the publication might have inadvertently documented a case of deer tick virus as a Powassan virus infection. Lastly, only articles published in the English language were evaluated; therefore, publications on Powassan virus outside of the US and Canada may have not been included.

## 5. Conclusions

In conclusion, Powassan virus encephalitis is associated with considerable mortality and poses a risk of long-term neurological complications. Clinicians should maintain a high index of suspicion for this disease in patients presenting with encephalitis, particularly those with a known or suspected tick exposure. Additionally, individuals who exhibit a combination of speech disorders, paralysis, ataxia, or cranial nerve dysfunction, along with basal ganglia lesions on imaging, should raise suspicion for Powassan virus. Given the increasing incidence and expanding geographic range of the virus, it is expected that the disease will become more prevalent in the coming years. Comprehensive clinical reporting, along with surveillance studies, and further research are warranted to better understand and respond to this emerging arbovirus and public health threat. Development of a vaccine against Powassan virus may became crucial in mitigating its impact on public health.

## Figures and Tables

**Table 1:** Inclusion and exclusion criteria for this study.

Inclusion Criteria	Exclusion Criteria
Studies providing detailed description of cases of Powassan virus infection	In vitro, in vivo, epidemiological studies, or reviews on Powassan virus
Cohort studies, case reports, and series were eligible for inclusion	Studies providing aggregate data on Powassan virus infections, without detailed descriptions of individual cases

**Table 3:** Prevalence of clinical manifestations of Powassan virus infections.

Finding	Adults (N = 70)	Children (N = 14)
**Symptoms**		
Fever	77.1%	100.00%
Headache	54.3%	50%
Confusion	42.9%	-
Fatigue	30%	15.40%
Nausea and Vomiting	24.3%	42.9%
Speech Disorders	32.9%	-
Visual Symptoms	27.1%	-
Hypersomnia and Lethargy	11.4%	42.9%
Myalgias and Arthralgias	8.6%	-
Memory Loss	5.7%	-
Seizures	1.4%	64.3%
**Signs**		
Altered Mental Status	50%	28.6%
Paresis	25.7%	14.3%
Cranial Nerve Involvement	25.7%	7.1%
Ataxia	24.3%	7.1%
Rash	14.3%	14.3%
Meningeal Signs	12.9%	23.10%
Tremors	10%	21.4%

**Table 4:** Findings of cerebrospinal fluid analysis.

Finding	Adults (N = 65)	Children (N = 14)
Median WBC (per mL)	119	137.5
Median Lymphocytes (per mL)	59.2	75.5
Median Neutrophils (per mL)	10.9	46
Mean Glucose (mg/dL)	64.9	65.3
Mean Protein (mg/dL)	88.2	35.5
Median Powassan Virus Antibody Titers	1:320	1:120

**Table 5:** Summary of MRI Findings.

Lesion Site	Adults (N = 56)	Children (N = 6)
Basal Ganglia	32.1%	50%
Cerebrum	26.8%	16.6%
Cerebellum	21.4%	-
Thalamus	14.3%	50%
Midbrain	8.9%	16.6%
Normal MRI	21.4%	33.3%

**Table 6:** Summary of outcomes.

Finding	Adults (N = 68)	Children (N = 14)
Mortality Rate	19.1%	7.1%
Paralysis	44.1%	42.6%
Cognitive Defects	33.3%	25%
Persistent Neurological Deficits	62.7%	50%

## Data Availability

All data pertaining to this study are presented in the current manuscript and its Supplementary Materials.

## Supplementary Materials

The following supporting information can be downloaded at https://www.mdpi.com/article/10.3390/tropicalmed8120508/s1, Supplementary Table S1: Murad scale for the evaluation on non-randomized trials; Supplementary Table S2: Evaluation of the quality of studies fulfilling the inclusion criteria by the Murad scale [13].

## Declarations

Part of the data presented in this manuscript were presented in the American College of Physicians Massachusetts Chapter Annual Scientific Meeting in September of 2023.
