# Supplementary material for: Powassan Virus Infections: A Systematic Review of Published Cases
Source: Trop Med Infect Dis. 2023 Nov 26;8(12):508. doi: 10.3390/tropicalmed8120508 (PMC10747444; doi:10.3390/tropicalmed8120508)
Supplement: Supplementary file 1 [file tropicalmed-08-00508-s001.zip › Kakoullis_Supplementary_Table_S1.pdf]

Supplementary Table S1: Murad scale for the evaluation on non-randomized trials[13].

Selection

1. Did the patient(s) represent all the cases of the medical center?

Ascertainment

2. Was the exposure adequately ascertained?
3. Was the outcome adequately ascertained?

Causality

4. Were other alternative causes that may explain the observation ruled out?
5. Was follow-up long enough for outcomes to occur?

Reporting

6. Is the case(s) described with sufficient details to allow other investigators to replicate the research or to allow practitioners make inferences related to their own practice?
