# Supplementary material for: Powassan Virus Infections: A Systematic Review of Published Cases
Source: Trop Med Infect Dis. 2023 Nov 26;8(12):508. doi: 10.3390/tropicalmed8120508 (PMC10747444; doi:10.3390/tropicalmed8120508)
Supplement: Supplementary file 1 [file tropicalmed-08-00508-s001.zip › Kakoullis_Supplementary_Table_S2.pdf]

Supplementary Table S2: Evaluation of the quality of studies fulfilling the inclusion criteria by the Murad scale[13]. Q1-Q6 are referring to the questions described in Supplementary Table S1.							
1st author, year	Selection	Ascertainment		Causality		Reporting	Total
	Q1	Q2	Q3	Q4	Q5	Q6	
McLean, 1959[2]	Yes	Yes	Yes	Yes	Yes	Yes	6
Goldfield, 1973[18]	Yes	Yes	Yes	Yes	Yes	Yes	6
Smith, 1974[19]	Yes	Yes	Yes	Yes	Yes	Yes	6
Rossier, 1974[20]	Yes	Yes	No	Yes	Yes	Yes	5
Wilson, 1979[21]	Yes	Yes	Yes	Yes	Yes	Yes	6
Partington, 1980[22]	Yes	Yes	Yes	Yes	Yes	Yes	6
Embil, 1983[23]	Yes	Yes	Yes	Yes	Yes	Yes	6
Fitch, 1990[24]	Yes	Yes	Yes	Yes	Yes	Yes	6
Gholam, 1999[25]	Yes	Yes	Yes	Yes	Yes	Yes	6
Courtney, 2001[26]	Yes	Yes	Yes	Yes	Yes	Yes	6
Lessell, 2003[27]	Yes	Yes	Yes	Yes	Yes	Yes	6
Hinten, 2008[6]	Yes	Yes	Yes	Yes	No	Yes	5
Tavakoli, 2009[28]	Yes	Yes	Yes	Yes	Yes	Yes	6
Trépanier, 2010[29]	Yes	Yes	Yes	Yes	Yes	Yes	6
Hicar, 2011[7]	Yes	Yes	Yes	Yes	Yes	Yes	6
Raval, 2012[30]	Yes	Yes	Yes	Yes	Yes	Yes	6
Choi, 2012[31]	Yes	Yes	Yes	Yes	Yes	Yes	6
Birge, 2012[4]	Yes	Yes	Yes	Yes	Yes	Yes	6
Sung, 2013[32]	Yes	Yes	Yes	Yes	Yes	Yes	6
Piantadosi, 2016[33]	Yes	Yes	Yes	Yes	Yes	Yes	6
Cavanaugh, 2017[34]	Yes	Yes	Yes	Yes	Yes	Yes	6
Tutolo, 2017[35]	Yes	Yes	Yes	Yes	Yes	Yes	6
Mittal, 2017[36]	Yes	Yes	Yes	Yes	Yes	Yes	6
Sanderson, 2018[37]	Yes	Yes	Yes	Yes	Yes	Yes	6
Solomon, 2018[38]	Yes	Yes	Yes	Yes	Yes	Yes	6
Patel, 2018[39]	Yes	Yes	Yes	Yes	Yes	Yes	6
Picheca, 2019[40]	Yes	Yes	No	Yes	No	Yes	4
Khan, 2019[41]	Yes	Yes	Yes	Yes	Yes	Yes	6
Allgaier, 2019[42]	Yes	Yes	Yes	Yes	Yes	Yes	6
Colman, 2020[43]	Yes	Yes	Yes	Yes	Yes	Yes	6
Koester, 2020[44]	Yes	Yes	Yes	Yes	Yes	Yes	6
Yu, 2020[45]	Yes	Yes	Yes	Yes	Yes	Yes	6
Feder, 2021[46]	Yes	Yes	Yes	Yes	Yes	Yes	6
Pach, 2021[47]	Yes	Yes	Yes	Yes	Yes	Yes	6
Dumic, 2021[48]	Yes	Yes	Yes	Yes	Yes	Yes	6
Dumic, 2021[49]	Yes	Yes	Yes	Yes	Yes	Yes	6
Taylor, 2021[50]	Yes	Yes	Yes	Yes	Yes	Yes	6
Kroopnick, 2021[51]	Yes	No	Yes	Yes	Yes	Yes	5
Nord, 2021[52]	Yes	No	Yes	Yes	Yes	No	4
Bazer, 2022[53]	Yes	Yes	Yes	Yes	No	Yes	5
Johnson, 2022[54]	Yes	Yes	Yes	Yes	Yes	Yes	6
Kakoullis, 2022[55]	Yes	Yes	Yes	Yes	Yes	Yes	6
Mendoza, 2023[56]	Yes	Yes	Yes	Yes	Yes	Yes	6
